# Non-invasive biofeedback-assisted vs. conventional pelvic floor muscle training combined with high-effort resistance training for urinary incontinence after radical prostatectomy: a randomized controlled trial

**DOI:** 10.1186/s12894-026-02333-9

**Published:** 2026-09-14

**Authors:** Marko Babović, David Aguayo, Joachim Wiskemann

**Affiliations:** 1 Institute of Sports and Sport Science, Heidelberg University, Heidelberg, Germany; 2 Working Group Exercise Oncology, Department of Medical Oncology, Heidelberg University Hospital and National Center for Tumor Diseases (NCT) Heidelberg, a partnership between DKFZ and University Medical Center Heidelberg, Im Neuenheimer Feld 460, Heidelberg, 69120 Germany; 3 Principal Scientist and Head of the Research Department of Kieser Training AG, Hardstraße 223, Zürich, 8005 Switzerland

**Keywords:** Prostate cancer, Urinary incontinence, Pelvic floor muscle training, Biofeedback, Resistance training

## Abstract

**Background:**

Radical prostatectomy (RP) often leads to urinary incontinence (UI). There is sufficient evidence from randomized controlled trials (RCTs) that pelvic floor muscle and sphincter training (PFMT) are effective interventions to reduce UI.

**Objective:**

To compare the effects of high-effort resistance training (HERT) combined with a non-invasive biofeedback-assisted device PFMT (dPFMT) versus HERT combined with conventional physiotherapeutic PFMT (pPFMT) on UI in prostate cancer patients undergoing RP.

**Methods:**

Men with a history of RP for localized prostate cancer were randomly assigned to dPFMT (*n* = 55) or pPFMT group (*n* = 54). Both PFMT intervention groups underwent a 12-week whole body HERT program performed at moderate to high loads (> 60% 1-repetition maximum, performed to momentary failure).

**Measurements:**

Urinary leakage was assessed with the 24-hour pad weight test (24-h pad test) at the baseline (T0) and at the end of the 12-week lasting programs (T1). Individual changes of cumulative pad weight in gram (g) from T0 to T1 were calculated and compared between groups. An UI reduction of ≥ 50% was considered as treatment success and compared between groups (primary endpoint).

**Results:**

Ninety-six participants (88%) completed the intervention. All participants demonstrated a statistically significant reduction in 24-h pad test values from T0 to T1 (Mean (SD): -86.39 g (336.25 g); 𝑝 < 0.001). Treatment success did not differ between groups (OR = 1.19, 95% CI 0.53–2.66; 𝑝 = 0.670) and was achieved by 52.1% of all participants. In a post hoc exploratory analysis restricted to participants achieving treatment success, the relative reduction in urinary leakage from T0 to T1 was greater in the dPFMT group than in the pPFMT group (mean (SD) = -83.97% (13.88%) vs. -74.68% (12.93%); 𝑝 = 0.017). This exploratory finding should be interpreted with caution, particularly because no adjustment for multiple testing was applied.

Time since surgery was a significant negative predictor of treatment success (*p* < 0.001), whereas age (*p* = 0.313) and follow-up rehabilitation treatment (AHB; *p* = 0.994) were not. None of the examined variables predicted the magnitude of change in urinary leakage.

**Conclusion:**

Both device-based and physiotherapist-guided PFMT were associated with significant reductions in objectively measured urinary leakage after RP. No statistically significant between-group differences were observed under conditions evaluated in this study.

**Trial registration:**

ClinicalTrials.gov Identifier NCT06206993, retrospectively registered on 15 December 2023.

**Supplementary Information:**

The online version contains supplementary material available at https://doi.org/10.1186/s12894-026-02333-9.

## Introduction

Prostate cancer remains a major global health concern, with approximately 1.4 million cases worldwide [1] and 79.600 new cases reported in Germany in 2023 [2]. Radical prostatectomy (RP), a standard treatment, is associated with postoperative urinary incontinence (UI) [3]. Although continence often recovers within months, 17–34% of patients report persistent UI 12 months post-surgery [4], significantly impairing quality of life (QoL) [5, 6]. Pelvic floor muscle training (PFMT) is recommended as an early conservative intervention following RP [7]. Randomized controlled trials (RCTs) have demonstrated that PFMT, whether conducted conventionally or biofeedback-assisted, accelerates continence recovery compared to no intervention [8]. Recent metaanalytic evidence further supports the beneficial effects of PFMT on continence recovery during the first six months following RP [9]. In patients treated more than 12 months post-RP, PFMT has been shown to reduce UI episodes by over 50% [10]. Meta-analytic evidence suggests biofeedback-assisted PFMT may provide additional benefits compared to conventional PFMT [11, 12]. Recent network meta-analytic evidence indicates that the relative effects of PFMT-based rehabilitation approaches may vary according to treatment modality and follow-up duration [13]. In addition to PFMT, resistance training (RT) has beneficial effects on physical function, muscle strength, and body composition in prostate cancer patients, as demonstrated in a meta-analysis by Keilani et al. [14]. Evidence indicates that RT improves physical performance, reduces fatigue, enhances QoL, and supports bone density, particularly during antihormonal therapy [15]. Moreover, higher physical activity levels are associated with reduced overall mortality (− 49%), cancer-specific mortality (− 61%), and disease progression (− 57%) [16]. Biofeedback-assisted PFMT has been introduced as a strategy to support training delivery. Non-invasive biofeedback-assisted systems, such as pelvic floor training machines designed for public or clinical settings, offer a standardized and non-invasive alternative to conventional approaches [11]. However, their effectiveness relative to physiotherapist-guided PFMT remains unclear. The Restoration of Continence (RECON) study therefore aimed to compare the effects of two 12-week interventions – device-based versus physiotherapist-led PFMT – on urinary incontinence in prostate cancer patients following RP.

## Materials and methods

### Design and participants

**Fig. 1:**
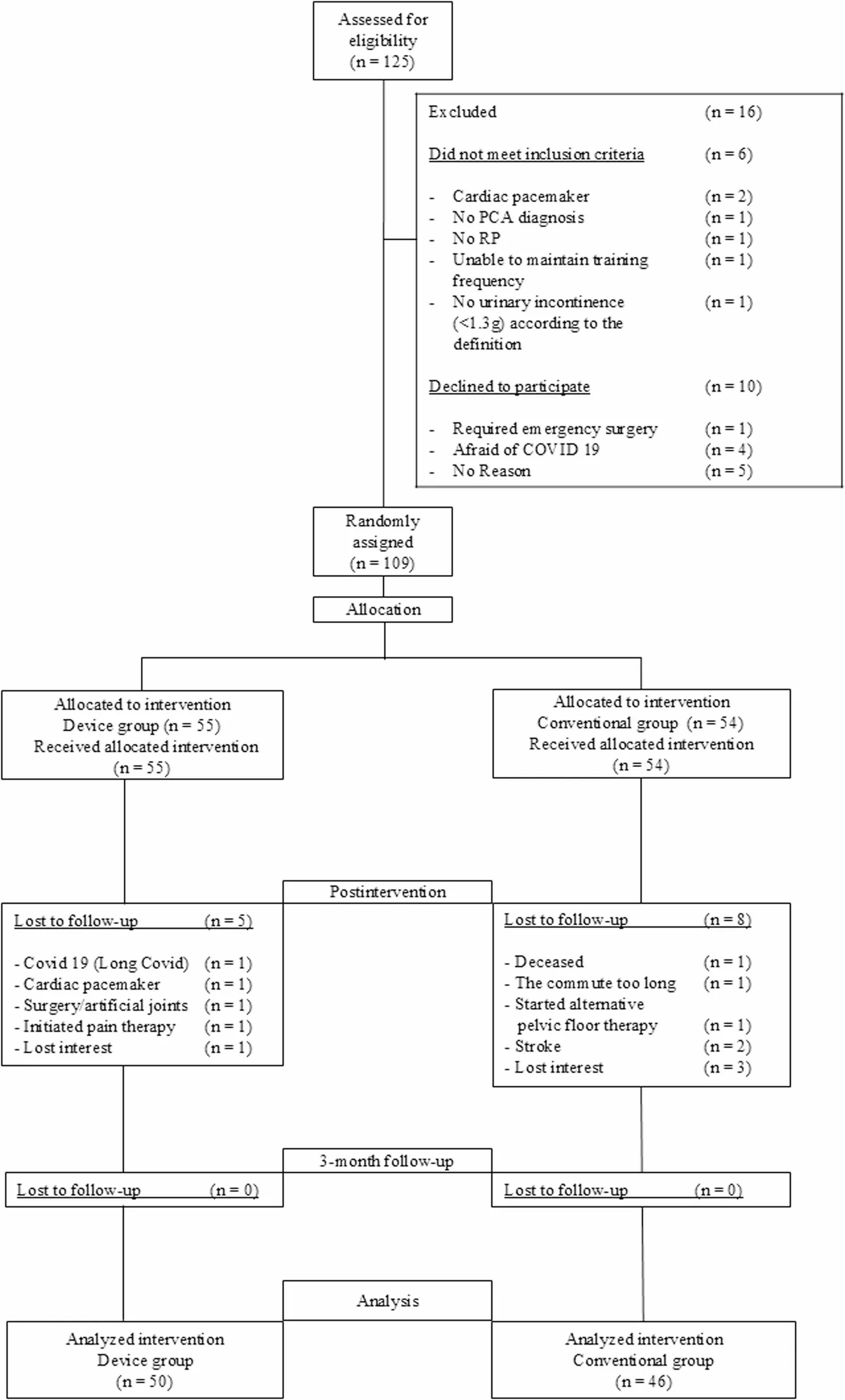
Participant flow chart through recruitment, randomization and intervention. Abbreviations: PCA, prostate cancer; RP, radical prostatectomy

The RECON study was a two-arm RCT investigating the effect of a 12-week high-effort RT program incorporating a non-invasive biofeedback-assisted PFMT device compared to a 12-week high-effort RT program supplemented by conventional PFMT with a physiotherapist on reducing UI among prostate cancer patients following RP. The study was initially designed and powered as a confirmatory non-inferiority randomized controlled trial (planned *n* = 180) with urinary continence (defined as zero pad use) as the primary endpoint, following the approach described by Overgard et al. [17]. During planning of the statistical analysis, and prior to any analysis of study outcomes, it became evident that this endpoint was not the most appropriate when using the standardized 24-hour pad test, which objectively quantifies urinary leakage by measuring pad weight gain. Because the objectively measured change in urinary leakage had already been predefined as a secondary outcome in the original study protocol, this objective outcome measure was adopted as the primary endpoint. Treatment success was accordingly defined as a clinically meaningful reduction in urinary leakage (≥ 50% reduction in 24-hour pad weight), based primarily on the work of Goode et al. [10]. Due to recruitment constraints during the COVID-19 pandemic, the study was terminated early after four years (August 2019–August 2023), with 109 participants enrolled. Additionally, contrary to the original protocol, many participants had long-standing UI rather than initiating treatment shortly after surgery. Participants were recruited in the Offenbach region (Germany) via hospitals, medical practices, and patient support networks. Eligible participants were men, aged 18 years or older with localized prostate cancer who underwent one of the following surgical procedures: radical retropubic prostatectomy (RRP), laparoscopic transperitoneal radical prostatectomy (LTRP), endoscopic extraperitoneal radical prostatectomy (EERP) and robotic assisted radical prostatectomy/DaVinci^®^ (RARP). Participants experiencing postoperative UI as a side effect were invited to participate in the trial between August 2019 and August 2023. Inclusion criteria comprised sufficient German language proficiency, ability to participate in supervised RT twice weekly for 12 weeks, and written informed consent. Exclusion criteria included unstable bone metastases; severe cardiac, neurological, pulmonary, or orthopedic conditions contraindicating RT; anorectal conditions contraindicating PFMT; prior radical perineal prostatectomy; neurological or muscular disorders affecting pelvic floor function; preoperative incontinence; neobladder; and pacemaker implantation. Figure 1 summarizes the actual reasons for exclusion among the screened participants, whereas the criteria listed above represent the predefined study eligibility criteria. Randomization was performed using minimization MS-DOS program Minim [18], stratified by surgical technique, baseline UI severity (≤ 200, 200–399, 400–599, 600–800, > 800 g), and age (≤ 65 vs. > 65 years). Randomization was performed by a sports scientist who was independent of the doctoral researcher. The doctoral researcher was not involved in the allocation process. After entry of the predefined stratification variables, treatment allocation was generated automatically by the minimization software according to the predefined allocation algorithm. The trial was retrospectively registered on 15 December 2023 at ClinicalTrials.gov (NCT06206993) and reviewed and approved on 01 July 2019 by the Ethics Committee of the Medical Faculty Heidelberg (S-458/2019). The study protocol had been finalized and submitted to the Ethics Committee before enrolment of the first participant and formed the basis for the ethics approval. This study was reported in accordance with the CONSORT 2010 statement.

### Intervention

Participants in both groups completed a 12-week supervised RT program twice weekly. The program consisted of nine machine-based exercises targeting major muscle groups (Supplementary Table 6) and was conducted using progressive, high-effort training (> 60% 1-repetition maximum [1-RM, performed to momentary failure]) [19]. This approach aligns with evidence supporting the benefits of RT for prostate cancer patients [4] and is consistent with the American College of Sports Medicine (ACSM) and exercise guidelines for cancer survivors and for the rehabilitation of patients with prostate carcinoma [20, 21]. Each session lasted approximately 60 minutes (min) and was supervised by trained exercise professionals. Prior to the intervention, all participants received standardized instruction on pelvic floor anatomy and correct muscle activation. For dPFMT group, RT was supplemented with a non-invasive biofeedback-assisted pelvic floor muscle training device (Kieser A5, Delphex Kräftigungstechnik GmbH, Breuberg, Germany). The device provided standardized training instructions through software and precise control of training intensity. Before each training session, participants performed three maximal voluntary pelvic floor contractions to determine their current maximum effort. Training intensity was then automatically adjusted to 80% of this individual maximum. During the standardized 104-second (s) training program, participants followed a predefined target curve consisting of 4-s-concentric, 2-s-isometric, 4-s-eccentric, and 2-s-relaxation phases, displayed as real-time graphical biofeedback. Training performance was quantified by the software according to the accuracy with which the predefined contraction pattern was followed. The device’s pressure-recording capability makes the process quantifiable, allowing effective monitoring and assessment of progress. During the exercise, participants followed a structured muscle contraction cycle displayed on a monitor. A digital log-in system recorded participants performance, enabling evaluation and progress tracking. Following the initial standardized instruction, participants performed the non-invasive biofeedback device training independently. During subsequent training sessions, qualified exercise professionals were available on the training floor to provide assistance when required, while the device’s real-time graphical biofeedback and standardized exercise protocol supported correct exercise execution and minimized compensatory movements. In the pPFMT group, participants performed conventional PFMT with a physiotherapist once weekly. Participants received individual instruction on pelvic floor anatomy and correct muscle contraction. Exercises were conducted in progressively more functional positions (supine, seated, all-fours position and upright) with each physiotherapy session lasting approximately 20–30 min, in accordance with established rehabilitation guidelines [22]. In addition, both groups were instructed to perform daily home-based pelvic floor muscle exercises in supine, seated and standing positions [4], progressing from 10 repetitions (5-s contraction/20-s rest) to 15 repetitions (10-s/15-s) and finally 15 repetitions (10-s/10-s) over the 12-week intervention.

### Blinding

Blinding was not feasible due to the nature of the PFMT interventions, as participants and supervising investigators could not be blinded to group allocation. Outcome assessment and data collection were standardized and conducted using objective measurement devices and dedicated software.

### Measurement and outcomes

The primary outcome was the change in urinary leakage from baseline (T0), prior to intervention, to the end of intervention period (T1). Urinary leakage was objectively measured using the 24-h pad test, expressed as the cumulative pad weight in grams (g). Based on related literature [10, 11], the intervention was considered successful if an individual demonstrated a reduction of UI of ≥ 50%. Adherence to the intervention programs was meticulously documented using exercise logs. Each participant received a unique login ID linked to an exercise log and documentation file, in which they recorded exercise duration, exercise types, and adherence to home-based sessions, RT sessions, PFMT, and, for the conventional group, physiotherapist-guided exercises. Reasons for missed sessions or early session termination, as well as subjective well-being, were also documented. As a cross-check, attendance lists were monitored, and the participants exercise logs were regularly reviewed.

### Statistical analyses

Baseline and group characteristics were analyzed using appropriate statistical methods. As change scores required data from T0 and T1, analyses were conducted based on available data from participants with complete outcome assessments at both time points. The primary endpoint was evaluated as an individual weight reduction in UI of ≥ 50%, based on prior benchmark [10, 11]. Treatment success rate was compared using the chi-square test. Normality of continuous variables was assessed using the Kolmogorov-Smirnov and Shapiro-Wilk test. For non-normally distributed data, within-group changes from T0 to T1 were analyzed using the Wilcoxon signed-rank test, while between-group comparisons were conducted using the Mann-Whitney U test. Binary logistic regression analysis was performed to identify predictors of treatment success. Age, time since surgery, and follow-up rehabilitation treatment were entered simultaneously as independent variables. Given the limited sample size, the exploratory regression models were kept parsimonious to reduce the risk of overfitting; additional variables, including surgical technique, were not included. Multiple linear regression analysis was conducted to examine predictors of change in 24-h pad weight from T0 to T1, using the same independent variables. Model assumptions were assessed prior to analysis. For logistic regression, model fit was evaluated using the Hosmer–Lemeshow test. For linear regression, residuals were inspected for normality and independence, and multicollinearity was assessed using variance inflation factors. All *p*-values reported are two-sided, with a significance level set at α = 0.05.

## Results

The baseline characteristics of the study population are summarized in Table 1, and the participant flow is illustrated in Fig. 1.

**Table 1:** Characteristics of the study population

Characteristics	No. of participants with data (*n* = 96)	dPFMT group (*n* = 50)	pPFMT group (*n* = 46)
Age (years), mean (range) [SD]	68.9 (53–84) [6.9]	68.6 (53–83) [6.9]	69.3 (56–84) [7.0]
≤ 65 years, n (%)	31 (32.3)	15 (30.0)	16 (34.8)
> 65 years, n (%)	65 (67.7)	35 (70.0)	30 (65.2)
Height (m), mean [SD]	1.78 [0.07]	1.79 [0.07]	1.78 [0.07]
Weight (kg), mean [SD]	83.9 [12.9]	81.6 [12.3]	86.5 [13.3]
BMI (kg/m²), mean [SD]	26.4 [3.8]	25.5 [3.6]	27.3 [3.9]
BMI category:			
Under-weight (< 18.5), n (%)	1 (1.0)	1 (2.0)	0 (0.0)
Normal-weight (18.5–24.9), n (%)	38 (39.6)	25 (50.0)	13 (28.3)
Over-weight (25–29.9), n (%)	42 (43.8)	17 (34.0)	25 (54.3)
Obese (≥ 30), n (%)	15 (15.6)	7 (12.0)	8 (17.4)
Surgical technique:			
Radical retropubic prostatectomy, n (%)	28 (29.2)	14 (28.0)	14 (30.4)
Laparoscopic transperitoneal radical prostatectomy, n (%)	22 (22.9)	13 (26.0)	9 (19.6)
Endoscopic extraperitoneal radical prostatectomy, n (%)	2 (2.1)	1 (2.0)	1 (2.2)
Robotic assisted radical prostatectomy/daVinci^®^, n (%)	44 (45.8)	22 (44.0)	22 (47.8)
Was follow-up rehabilitation treatment (AHB) taken:			
Yes, n (%)	72 (75.0)	36 (72.0)	36 (78.3)
No, n (%)	24 (25.0)	14 (28.0)	10 (21.7)
Time since diagnosis(months), mean [SD]	34.5 [47.8]	33.0 [45.2]	36.1 [50.9]
Time since surgery, (months), mean [SD](range)	29.5 [47.5] (< 1 to 244 months)	28.4 [44.2] (< 1 to 200 months)	30.7 [51.4] (< 1 to 244 months)
24-h pad test, pads/day, mean (range) [SD]	3.7 (1–11) [1.9]	3.6 (1–11) [2.0]	3.7 (1–8) [1.8]
24-h pad test, urinary leakage (g), mean (range) [SD]	244.5 (2.3–1785.0) [374.1]	234.7 (2.3-1260.4) [350.9]	255.1 (2.9–1785.0) [401.5]
Severe incontinence:	16 (16.7)	7 (14.0)	9 (19.6)
> 450 g per 24 h, n (%)			
Heavy incontinence:	19 (19.8)	10 (20.0)	9 (19.6)
200–450 g per 24 h, n (%)			
Moderate incontinence:	13 (13.5)	10 (20.0)	3 (6.5)
50–200 g per 24 h, n (%)			
Mild incontinence:	48 (50.0)	23 (46.0)	25 (54.3)
<50 g per 24 h, n (%)			

Data are presented as mean [standard deviation] or number (%), unless otherwise indicated

*Abbreviation*: *BMI* Body mass index, which is calculated as weight in kilograms divided by height in meters squared, *AHB *Anschlussheilbehandlung; follow-up rehabilitation

**Fig. 2:**
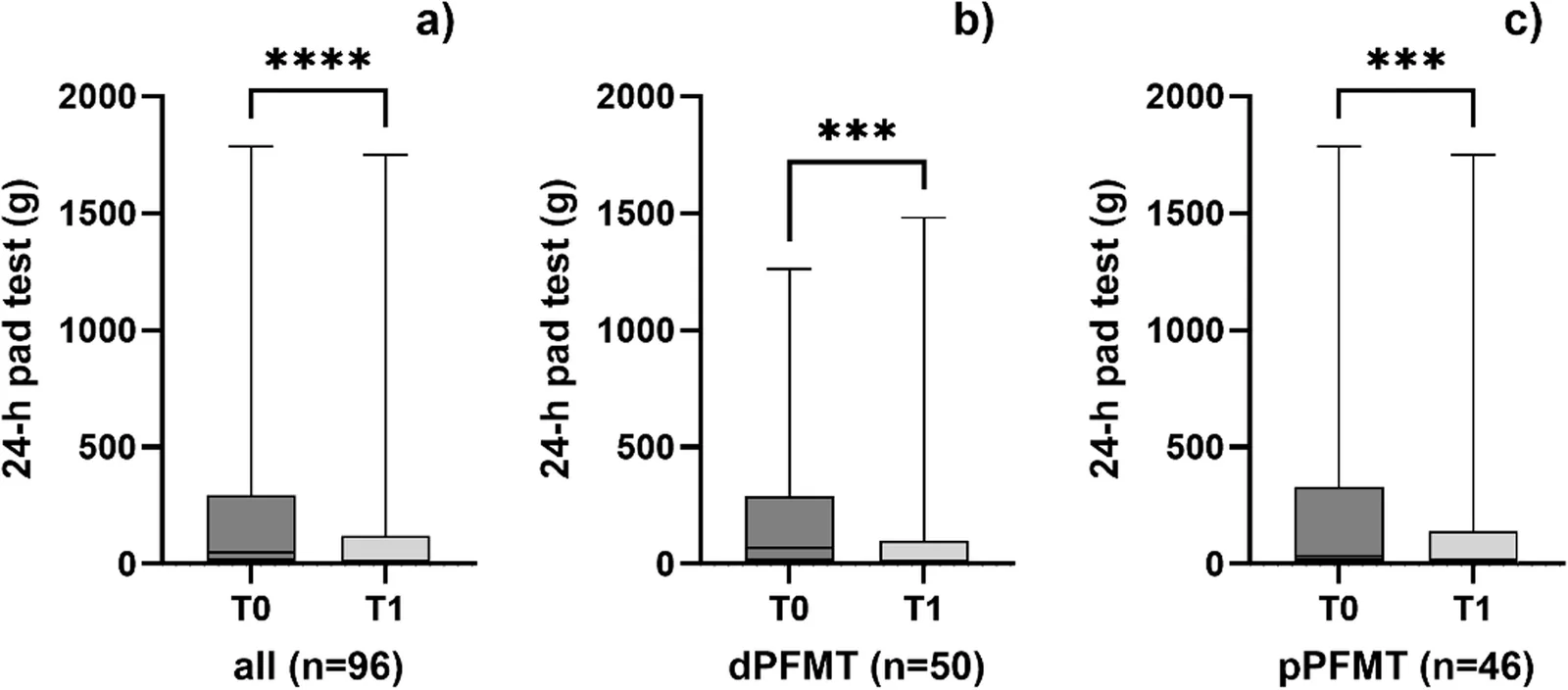
24-h pad test results at T0 and T1 for (**a**) all partcipants ( *n *= 96), (**b**) the dPFMT group (*n* = 50), and (**c**) the pPFMT group (*n* = 46). Abbreviations: T0, baseline; T1, post-intervention; dPFMT, device-assisted pelvic floor muscle training; pPFMT, physiotherapist-guided pelvic floor muscle training. Boxes represent the interquartile range with median values indicated by the horizontal line; whiskers denote the full data range. Significant reductions in 24-h pad test from T0 to T1 were observed in all (*n* = 96) groups; Wilcoxon matched-pairs signed-rank test; **** *p* < 0.0001 for all, *** *p* < 0.001 for dPFMT (*n* = 50) and pPFMT (*n* = 46)

Of 125 men screened, 109 who had undergone RP for clinically localized prostate cancer and experienced postoperative UI were included in this RCT, which was conducted from August 2019 to August 2023. The cohort had a mean age of 68.9 years (SD 6.9 years), with no between-group differences. Mean BMI was 26.4 kg/m² (SD 3.8 kg/m²), with a higher BMI in the pPFMT group. Surgical techniques and AHB participation were comparable. Baseline urinary leakage was 244.5 g (SD 374.1 g), with a mean pad use of 3.7 (SD 1.9) pads/day (Table 1). The mean time since surgery was 29.53 months (SD 47.54 months; median: 7 months). Of the 109 participants, 96 (88%) completed the intervention (dropout rate: 12%), primarily due to loss of interest (Fig. 1). Adherence among completers was 100% for supervised sessions (24/24) and scheduled physiotherapy sessions (12/12). Analyses were conducted in participants with available outcome data. Treatment success (*≥* 50% UI reduction) was achieved by 52.1% of participants, with no significant between-group difference (dPFMT: 50%; pPFMT: 54.3%; OR = 1.19, 95% CI 0.53–2.66; χ²(1) = 0.18, *p* = 0.670; Table 3). Urinary leakage decreased significantly from T0 to T1 across all participants (*p* < 0.001), with an average reduction of -21.62% (SD 112.81%) or -86.39 g (SD 336.25 g). No significant between-group differences were observed; however, both groups showed significant within-group improvements (dPFMT: -110.16 g (SD 344.42 g), *p* = 0.001; pPFMT: -60.56 g (SD 328.96 g), *p* < 0.001; Fig. 2).

**Fig. 3:**
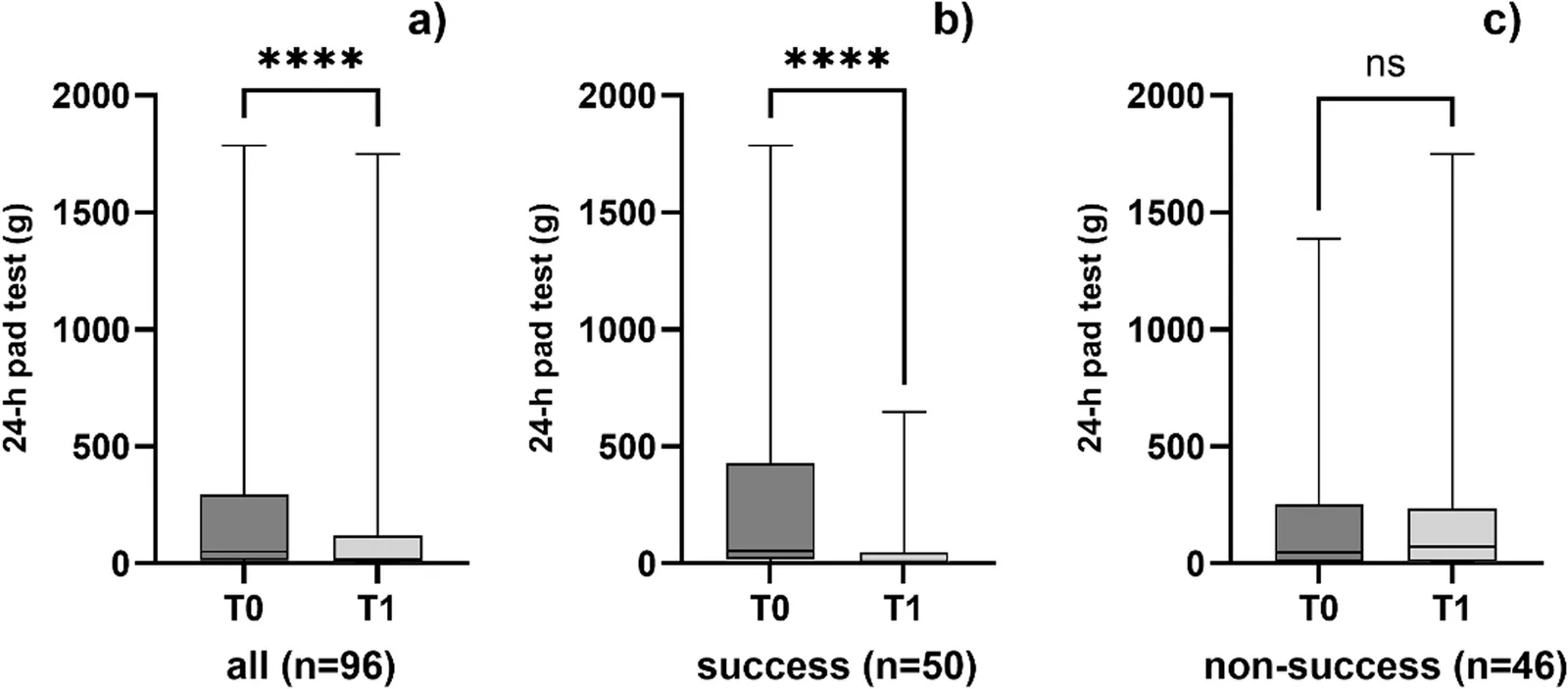
24-h pad test results by treatment success status at T0 and T1 for (**a**) all participants (*n* = 96), (**b**) the success group (*n* = 50), and (**c**) the non-success group (*n* = 46). Abbreviations: T0, baseline; T1, post-intervention; ns, not significant. Boxes represent the interquartile range with median values indicated by the horizontal line; whiskers denote the full data range. Significant reductions in 24-h pad test from T0 to T1 were observed in all (*n* = 96) and in the success group (*n* = 50) (Wilcoxon matched-pairs signed-rank test; **** *p* < 0.0001), whereas no significant change was detected in the non-success group (*n* = 46); (ns, *p* ≥ 0.05)

In a post hoc exploratory analysis of participants who achieved treatment success, a significant between-group difference was observed (-83.97% (SD 13.88%) vs. -74.68% (SD 12.93%), *p* = 0.017), favoring the dPFMT group (Table 2). Conversely, among participants in the non-success group, the mean relative change in cumulative pad weight from T0 to T1 was 41.1% (SD 137.6%) overall, with 29.2% (SD 79.6%) in the dPFMT group and 55.3% (SD 186.0%) in the pPFMT group, with no between-group difference (*p* = 0.168). Within-group comparisons for the non-success group revealed no significant changes from T0 to T1 for either the dPFMT (*p* = 0.412) or the pPFMT group (*p* = 0.821). Figure 3 illustrates the distribution of 24-h pad test values at T0 and T1 for the total population and both groups by treatment success. The minimum expected cell frequency was 22.04, indicating no violation of chi-square test assumptions (Table 3).

**Table 2:** Relative change in cumulative weight (g) from T0 to T1 in % for the success group, i.e. urinary incontinence reduction ≥ 50%

Relative change in cumulative weight (g) from T0 to T1 in % for the success group, i.e. urinary incontinence reduction ≥ 50%					
Group	Reduction *≥* 50% (*n* = 50)	Mean (SD)	Median (IQR)	*p*-value	Intergroup difference *p*-value
All	50	-79.32 (14.1)	-81.01	< 0.001	0.017 favoring device group
RT + PFMT (Device group)	25	-83.97 (13.9)	-90.56	< 0.001	
RT + PFMT (Conventional group)	25	-74.68 (12.9)	-78.56	< 0.001	

RT = Resistance Training; PFMT = Pelvic Floor Muscle Training

T0 = Baseline; T1 = Post-intervention

Success = ≥ 50% reduction in 24-h pad test

*p*-values < 0.05 considered statistically significant

Intergroup comparisons refer to the difference between device and conventional groups

Data are presented as Mean (SD) or Median (IQR), unless otherwise indicated

**Table 3:** Treatment success rates and change in UI from T0 to T1, assessed by the 24-h pad test in all, dPFMT and pPFMT groups

All Participants - Relative change from T0 to T1 in %								
Group	Mean (SD)			Median (IQR)	*p*-value			
All (*n* = 96)	-21.62 (112.8)			-53.05	< 0.001			
RT + dPFMT (*n* = 50)	-27.39 (80.4)			-51.98	0.001			
RT + pPFMT (*n* = 46)	-15.35 (140.5)			-53.05	< 0.001			

Treatment success based on the primary endpoint: Urinary incontinence reduction ≥ 50%								
Group	Success (*n* / %)		Non-Success (*n* / %)	Overall (*n* / %)	x²	df	OR (95% CI)	*p*-value
RT+ dPFMT	25 (50.0)		25 (50.0)	50 (100)	0.18	1	Reference	0.670
RT + pPFMT	25 (54.3)		21 (45.7)	46 (100)			1.19 (0.53–2.66)	
Overall	50 (52.1)		46 (47.9)	96 (100)				

cumulative weight (g)								
	at T0	at T1		Total change from T0 to T1				
Group	Mean (SD)	Mean (SD)		Mean (SD)	Median (IQR)		*p*-value	
All (*n* = 96)	244.5 (374.1)	158.1 (351.5)		-86.39 (336.2)	-14.79		< 0.001	
RT + dPFMT (*n* = 50)	234.7 (350.9)	124.6 (270.2)		-110.16 (344.4)	-15.51		0.001	
RT + pPFMT (*n* = 46)	255.1 (401.5)	194.6 (422.7)		-60.56 (329.0)	-14.10		< 0.001	

RT = Resistance Training; PFMT = Pelvic Floor Muscle Training

T0 = Baseline; T1 = post-intervention

Success = ≥ 50% reduction in 24-h pad test

*p*-values < 0.05 considered statistically significant

Intergroup comparisons refer to the difference between device and conventional groups

Data are presented as Mean (SD) or Median (IQR), unless otherwise indicated

OR = odds ratio; CI = confidence interval; dPFMT served as the reference group for the between-group comparison

To identify predictors of treatment outcome, binary logistic and multiple linear regression analyses were conducted, including age, time since surgery, and participation in AHB. In the binary logistic regression model, time since surgery emerged as a statistically significant predictor of treatment success (B = − 0.045, SE = 0.013, *p* < 0.001, OR = 0.956, 95% CI 0.932–0.981). Increasing time since surgery was associated with lower odds of treatment success. In contrast, neither age (*p* = 0.313) nor participation in AHB (*p* = 0.994) significantly predicted treatment success. The overall logistic regression model was statistically significant (χ² = 27.83, *p* < 0.001), indicating that the predictors reliably distinguished between patients with and without treatment success. Model fit was satisfactory, as evidenced by a non-significant Hosmer–Lemeshow test (*p* = 0.932). The model explained 33.6% of the variance in treatment success (Nagelkerke R² = 0.336, Table 4).

**Table 4:** Predictors of treatment outcome - Binary logistic regression predicting treatment success

Predictor	B	SE	*p*-value	OR (Exp(B))	95% CI
Age	0.037	0.036	0.313	1.037	0.966–1.114
Time since surgery (months)	−0.045	0.013	< 0.001	0.956	0.932–0.981
AHB (yes vs. no)	−0.005	0.573	0.994	0.995	0.324–3.060
Model fit: χ² = 27.83, *p* < 0.001 Nagelkerke R² = 0.336 Hosmer–Lemeshow *p* = 0.932					

*Abbreviation*: *AHB *Anschlussheilbehandlung; follow-up rehabilitation

*p*-values < 0.05 considered statistically significant

While time since surgery was a significant predictor of treatment success in the logistic regression model, it was not associated with the magnitude of change in urinary leakage. This discrepancy may be explained by the dichotomous definition of treatment success and the high interindividual variability in continuous outcome measures. To examine potential predictors of the magnitude of change in urinary leakage, a multiple linear regression analysis was conducted, using the change in 24-h pad test from baseline to post-intervention as the dependent variable. The overall regression model was not statistically significant (F(3,92) = 1.19, *p* = 0.317, adjusted R² = 0.006). None of the independent variables were significant predictors of the magnitude of change in urinary leakage (age, *p* = 0.519; time since surgery, *p* = 0.158; AHB, *p* = 0.734; Table 5).

**Table 5:** Predictors of treatment outcome - Multiple linear regression predicting change in urinary leakage

Predictor	B	SE	β	*p*-value
Age	3.387	5.235	0.070	0.519
Time since surgery (months)	1.083	0.761	0.153	0.158
AHB	*11.088*	*32.529*	*0.035*	*0.734*
Model: F(3,92) = 1.19, *p* = 0.317 Adjusted R² = 0.006				

*Abbreviation*: *AHB *Anschlussheilbehandlung; follow-up rehabilitation

*p*-values < 0.05 considered statistically significant

No adverse events related to the intervention were observed or reported.

## Discussion

This randomized controlled RECON trial demonstrated that PFMT, whether performed using a non-invasive biofeedback-assisted device (dPFMT group) or as conventional PFMT (pPFMT group), and combined with a high-effort, full-body RT training program performed twice per week, effectively reduced UI in prostate cancer patients after RP. Across the cohort, mean UI was reduced by 21.62% at the end of the intervention. No statistically significant between-group difference was detected. However, as the study was not designed as a formal non-inferiority or equivalence trial, the absence of a statistically significant between-group difference should not be interpreted as evidence of equivalence. Clinically meaningful symptomatic improvements occurred in both groups; however, treatment success rates (defined as ≥ 50% reduction in UI) did not differ significantly. Although the pPFMT group had a higher mean BMI at baseline, no further statistical analyses were performed. Randomization was conducted independently of BMI using the pre-specified stratification factors. In accordance with CONSORT 2010 guidelines, formal hypothesis testing of baseline differences was deliberately omitted. Specifically, 50% of participants in the dPFMT group (25/50) and 54.3% in the pPFMT group (25/46) achieved treatment success. A post hoc exploratory analysis of participants who achieved treatment success revealed that participants in the dPFMT group exhibited a 9.29% greater UI reduction (-83.97%) compared to participants in pPFMT group (-74.68%; 𝑝 = 0.017). As this was a post hoc exploratory analysis and no adjustment for multiple testing was applied, these findings should be interpreted with caution and require confirmation in future prospectively designed studies with a pre-specified analysis plan. The pPFMT intervention represented a standardized, physiotherapist-guided approach based on established rehabilitation recommendations. However, PFMT intensity, frequency, and individualization may vary across specialized rehabilitation settings; thus, the present pPFMT protocol may not fully represent all forms of specialized pelvic floor physiotherapy. Both groups received the same twice-weekly RT program and daily home-based PFMT. The additional PFMT exposure differed between groups: the dPFMT group performed the standardized 104-s device-assisted PFMT twice weekly, whereas the pPFMT group received one 20–30-min physiotherapist-guided PFMT session per week. Consequently, the interventions differed in the frequency and duration of additional PFMT exposure, which should be considered when interpreting between-group comparisons. These results align with previous literature demonstrating that PFMT reduces urinary leakage after RP, frequently achieving ≥ 50% reduction in UI [10, 11]. RT was included based on evidence demonstrating improvements in physical performance and muscle strength, in prostate cancer patients [14], consistent with established exercise guidelines for cancer survivors [20, 21]. However, evidence for a direct effect of RT on urinary continence recovery after RP remains limited, and such an effect cannot be inferred from the present study. As RT was applied equally across both study arms, it likely contributed to overall improvements but did not confound group comparisons. Importantly, because both intervention groups received the same HERT program and no RT-only control group was included, the independent contributions of PFMT and RT to the observed improvements cannot be determined. Therefore, the present findings should be interpreted as reflecting the combined intervention rather than the isolated effects of either component. The cohort (mean age 69 years) was representative of the target population [1]. Age was not associated with UI reduction or treatment success, contrasting with recent evidence indicating poorer continence recovery with increasing age [23]. Greco et al. reported similar continence rates across age groups at 1, 3, and 12 months postoperatively [24]. The mean time from surgery to intervention in our cohort was 29.5 months, indicating that measurable improvements can be achieved even in patients with long-standing or chronic UI. The time elapsed between surgery and intervention initiation emerged as a potentially important factor. Regression analysis indicated that a shorter time since surgery was associated with a higher likelihood of achieving a clinically meaningful treatment response (≥ 50% reduction); whereas no consistent association was observed for the absolute magnitude of leakage reduction. Although the observed monthly effect was relatively small, the findings suggest that earlier initiation of rehabilitation may be associated with a higher probability of treatment success. Because time since surgery was analyzed as a continuous variable, the present data do not allow the identification of a clinically meaningful threshold. At the same time, the broad range in time since surgery reflects the heterogeneous population encountered in routine clinical practice and may therefore enhance the generalizability of the findings. However, this heterogeneity may also have contributed to variability in treatment response, potentially reducing the ability to detect modest differences between the intervention groups. These findings are consistent with recent evidence suggesting that early initiation of PFMT may facilitate continence recovery after RP [25]. Continuous outcome measures, such as 24-h pad test reduction, may exhibit substantial interindividual variability [26]. Moreover, continence recovery after RP is influenced by multiple patient-, anatomical-, and treatment-related factors [27], which may contribute to variability in treatment response. Recent meta-analytic evidence suggests that the relative effects of PFMT-based rehabilitation modalities may vary across postoperative follow-up periods [13]. Consistent with these findings, both interventions in the present study were associated with improvements even in participants treated more than 12 months postoperatively. Although our post hoc exploratory responder analysis suggests that selected patients may still derive additional benefit from biofeedback-assisted PFMT, the clinical importance of this difference remains to be identified. The greater reduction observed in dPFMT responders may be explained by enhanced neuromuscular activation and motor learning facilitated by real-time biofeedback [11]. Evidence on postoperative interventions beyond 12 months remains limited, particularly for combined biofeedback-assisted PFMT with structured RT [28]. Overall, both conventional and device-assisted PFMT were associated with improvements in post-RP UI, including in patients with long-standing UI. The post hoc exploratory responder analysis suggests a potential additional benefit of non-invasive biofeedback-assisted PFMT in selected responders.

### Limitations and strengths

Several limitations must be acknowledged. The primary endpoint was modified before the statistical analyses were performed. Although this decision was based on methodological considerations and involved an outcome measure that had already been predefined in the original study protocol, this modification limits the confirmatory interpretation of the study findings. Accordingly, the results should be interpreted as exploratory rather than confirmatory. The trial was retrospectively registered at ClinicalTrials.gov after participant recruitment had been completed. However, the complete study protocol had been finalized before recruitment commenced and was submitted to the Ethics Committee of the Medical Faculty Heidelberg as part of the ethics approval process. Ethical approval (S-458/2019) was obtained before enrolment of the first participant. Therefore, the study design and planned study procedures were predefined before study initiation. Nevertheless, retrospective trial registration may reduce methodological transparency and should therefore be considered when interpreting the findings. Recruitment took place during the COVID-19 pandemic, when cancer patients were classified as high-risk and public health restrictions limited enrollment, resulting in a smaller sample size than planned. Consequently, the reduced sample size may have limited the statistical power to detect modest between-group differences. Because participants without post-intervention measurements could not be included in the primary outcome analysis, the primary analysis was based on complete cases, which should be considered when interpreting the findings. Although a follow-up assessment (T2) was performed 12 weeks after completion of the intervention, these data were beyond the scope of the present manuscript, which focused on the primary endpoint assessed immediately after completion of the intervention (T1). Blinding was not feasible due to the nature of the interventions. To minimize bias, a predefined study protocol, objective outcome assessment, and independent assessments were applied. The 24-h pad test was subsequently selected as the primary endpoint in line with International Consultation on Incontinence recommendations, emphasizing objective over self-reported measures. Self-reported pad use was not considered sufficiently accurate for quantifying UI severity, due to its limited accuracy in capturing urinary leakage [29]. The 24-h pad test is widely regarded as the gold standard for objective and reproducible assessment of UI severity in clinical research [26, 28]. Although patient-reported outcomes, including quality-of-life measures, were prospectively collected as part of the RECON study, these outcomes were beyond the scope of the present analysis. Consequently, the present findings do not allow objective changes in urinary leakage to be directly related to patient-reported changes in quality of life or perceived symptom burden. A significant strength of this study was the use of a biofeedback device with integrated software, enabling standardized instruction and precise training adjustments. Real-time graphical feedback enabled participants to visualize muscle contractions, improving both motor learning and engagement. While adherence to supervised sessions was excellent—with 100% completion rates for PFMT and physiotherapy—adherence to home-based exercises was self-reported and remains subject to potential reporting bias.

### Clinical implications and future directions

The RECON trial showed significant reductions in UI following postoperative care combining PFMT with HERT in prostate cancer patients. Both dPFMT and pPFMT approaches were associated with improvements in UI, with no statistically significant difference in overall treatment success under the conditions evaluated in this study. However, the post hoc exploratory responder analysis suggests a potential additional benefit of biofeedback-assisted PFMT in selected responders. The non-invasive device offers practical advantages, including standardized application without invasive components and reduced reliance on specialized personnel. This may be particularly relevant in the context of increasing rehabilitation demand and workforce constraints, supporting scalable and resource-efficient care models [30, 31]. Integration into supervised training programs enables implementation without additional burden and may facilitate transition to independent exercise. From a health economic perspective, biofeedback-assisted approaches may reduce long-term costs associated with persistent UI [32] and support faster functional recovery, potentially facilitating earlier return to daily activities. Future research should focus on the long-term effectiveness of these intervention outcomes, particularly for patients initiating treatment 12 months or more after surgery. Further investigation into optimal timing, patient-specific factors (e.g., age, comorbidities, surgical approach), and the potential synergistic effects of combining structured RT with biofeedback is warranted to further refine postoperative rehabilitation strategies.

## Conclusion

In this randomized controlled trial, both device-based PFMT (dPFMT) and conventional PFMT (pPFMT), when combined with a high-effort, full-body RT program, were associated with significant reductions in objectively measured urinary leakage following RP. No statistically significant between-group difference was observed under the conditions evaluated in this study. The findings of the post hoc exploratory subgroup analysis should be considered hypothesis-generating and require confirmation in future prospectively designed studies.

## Supplementary Information

Supplementary Material 1.

Supplementary Material 2.

## Data Availability

The datasets generated and/or analyzed during the current study are available from the corresponding author on reasonable request.

## Abbreviations

ACSM: American College of Sports Medicine
AHB: Anschlussheilbehandlung (follow-up rehabilitation)
BMI: Body mass index
CI: Confidence interval
dPFMT: Device-assisted pelvic floor muscle training
EERP: Endoscopic extraperitoneal radical prostatectomy
HERT: High-effort resistance training
IQR: Interquartile range
LTRP: Laparoscopic transperitoneal radical prostatectomy
OR: Odds ratio
PCA: Prostate cancer
PFMT: Pelvic floor muscle training
pPFMT: Physiotherapist-guided pelvic floor muscle training
QoL: Quality of life
RARP: Robotic-assisted radical prostatectomy
RCT: Randomized controlled trial
RECON: Restoration of Continence
RP: Radical prostatectomy
RRP: Radical retropubic prostatectomy
RT: Resistance training
SD: Standard deviation
SE: Standard error
T0: Baseline
T1: Post-intervention
T2: 12-week follow-up
UI: Urinary incontinence
1-RM: One-repetition maximum
