# Supplementary material for: Non-invasive biofeedback-assisted vs. conventional pelvic floor muscle training combined with high-effort resistance training for urinary incontinence after radical prostatectomy: a randomized controlled trial
Source: BMC Urol. 2026 Sep 14;26:219. doi: 10.1186/s12894-026-02333-9 (PMC13584499; doi:10.1186/s12894-026-02333-9)
Supplement: Supplementary file 1 [file 12894_2026_2333_MOESM2_ESM.docx]

| **Strength-Training**  **Exercise machines** | **Exercise description and muscles** |
| --- | --- |
| A3 | Abductor – small and middle gluteal muscles (m.glutaeus minimus and m.glutaeus medius) |
| B1 | Leg Extension – quadriceps muscle of the thigh (m.quadriceps) |
| B7 | Seated leg curl – hamstring muscles (m.biceps femoris and m.semitendinosus) |
| F2.1 | Abdominal – straight muscle of abdomen (m.rectus abdominis) |
| F1.1 | Rotary Torso – oblique muscles of the abdomen plus oblique section of the deep spinal erectors (mm.obliqui abdomini) |
| F3.1 | Lower back – erector muscle of spine / Autochthonous back muscle (m.erector spinae) |
| C1 | Pullover – latissimus dorsi muscle (m.latisimus dorsi) |
| D6 | Chest press – greater pectoral, deltoid muscle front section and triceps muscle of arm (m pectoralis major, m.deltoideus and m.triceps brachii) |
| E2 | Lateral raise – deltoid muscle middle section and trapezius muscle upper section (m.deltoideus and m.trapezius), |

Table 6. Exercise machines and description
